# Continuous Multimodality Monitoring in Children after Traumatic Brain Injury—Preliminary Experience

**DOI:** 10.1371/journal.pone.0148817

**Published:** 2016-03-15

**Authors:** Adam M. H. Young, Joseph Donnelly, Marek Czosnyka, Ibrahim Jalloh, Xiuyun Liu, Marcel J. Aries, Helen M. Fernandes, Matthew R. Garnett, Peter Smielewski, Peter J. Hutchinson, Shruti Agrawal

**Affiliations:** 1 Division of Academic Neurosurgery, Department of Clinical Neurosciences, Addenbrooke's Hospital, University of Cambridge, Cambridge, United Kingdom; 2 Department of Pediatric Intensive Care, Addenbrooke's Hospital, University of Cambridge, Cambridge, United Kingdom; 3 Institute of Electronic Systems, Warsaw University of Technology, Poland; 4 Department of Intensive Care, University of Groningen, University Medical Center Groningen, The Netherlands; University of Pennsylvania, UNITED STATES

## Abstract

**Introduction:**

Multimodality monitoring is regularly employed in adult traumatic brain injury (TBI) patients where it provides physiologic and therapeutic insight into this heterogeneous condition. Pediatric studies are less frequent.

**Methods:**

An analysis of data collected prospectively from 12 pediatric TBI patients admitted to Addenbrooke’s Hospital, Pediatric Intensive Care Unit (PICU) between August 2012 and December 2014 was performed. Patients’ intracranial pressure (ICP), mean arterial pressure (MAP), and cerebral perfusion pressure (CPP) were monitored continuously using brain monitoring software ICM+^®^,) Pressure reactivity index (PRx) and ‘Optimal CPP’ (CPPopt) were calculated. Patient outcome was dichotomized into survivors and non-survivors.

**Results:**

At 6 months 8/12 (66%) of the cohort survived the TBI. The median (±IQR) ICP was significantly lower in survivors 13.1±3.2 mm Hg compared to non-survivors 21.6±42.9 mm Hg (p = 0.003). The median time spent with ICP over 20 mm Hg was lower in survivors (9.7+9.8% vs 60.5+67.4% in non-survivors; p = 0.003). Although there was no evidence that CPP was different between survival groups, the time spent with a CPP close (within 10 mm Hg) to the optimal CPP was significantly longer in survivors (90.7±12.6%) compared with non-survivors (70.6±21.8%; p = 0.02). PRx provided significant outcome separation with median PRx in survivors being 0.02±0.19 compared to 0.39±0.62 in non-survivors (p = 0.02).

**Conclusion:**

Our observations provide evidence that multi-modality monitoring may be useful in pediatric TBI with ICP, deviation of CPP from CPPopt, and PRx correlating with patient outcome.

## Introduction

Traumatic brain injury (TBI) remains a major public health problem, particularly in children. Epidemiological studies demonstrate that the incidence of hospitalisation and fatal TBI is disproportionately high in children. The Centre for Disease Control and Prevention report over 1.4 million incidents of TBI in children and in excess of 50,000 deaths in the US alone [1]. Despite this, little is known about the pathophysiology of acute brain injury in children [2].

Multi-modal brain monitoring has been advised to guide management of severe TBI in adults [3] however, there is limited experience with advanced brain monitoring in pediatrics, although some pioneering studies have been published [4,5]. Multi-modal monitoring affords clinicians an early indication of secondary insults to the recovering brain by identifying features such as raised intracranial pressure (ICP) and decreased cerebral perfusion pressure (CPP; defined as mean arterial pressure minus ICP). Although there is some consensus as to what value of ICP is unacceptably high, the optimal level of CPP is more contentious [6]. This is particularly pertinent in the pediatric population where the ‘normal’ mean arterial pressure (MAP) is age dependent and critical levels of ICP are not well characterized.

Cerebrovascular pressure reactivity indicates how ICP changes in relation to changes in MAP and can be assessed using the pressure reactivity index (PRx). PRx is calculated as a moving, linear correlation between slow waves of MAP and ICP, and can be considered an estimator of cerebral autoregulation [7]. A negative PRx indicates intact pressure reactivity, whereas a positive PRx indicates impaired pressure reactivity. Using PRx, a method for finding the CPP at which the vasculature is most reactive has been proposed [8,9]. By plotting the average PRx across different ranges of CPP, the CPP with the most negative (or best) PRx can be depicted automatically as a continuous time-dependent variable—the ‘optimal’ CPP (CPPopt) [10].

In pediatric TBI, patients with a lower PRx have a greater chance for survival [5]. In that cohort, PRx was dependent on the CPP level; a finding that raises the possibility of defining CPPopt targets for children. Here, we describe multimodality monitoring of children who have sustained a severe traumatic brain injury.

## Methodology

### Patients

The data in this study were collected prospectively from 12 pediatric TBI patients admitted to Addenbrooke’s Hospital, Cambridge, Pediatric Intensive Care Unit (PICU) between August 2012 and December 2014. Consecutive TBI patients with a clinical need for ICP monitoring were included for analysis. The insertion of an intracranial monitoring device is part of routine clinical practice and as such did not require ethical approval. The data is routinely collected for clinical purposes and guides the management of patients. The analysis of data within this study for the purposes of service evaluation was approved by the Cambridge University Hospital NHS Trust, Audit and Service Evaluation Department (Ref:2143) and did not require ethical approval or patient consent. The treatment of patients was guided purely based on ICP and CPP measurements, the indices of PRx and CPPopt were observed only and did not determine the management of the cohort.

**Fig 1:**
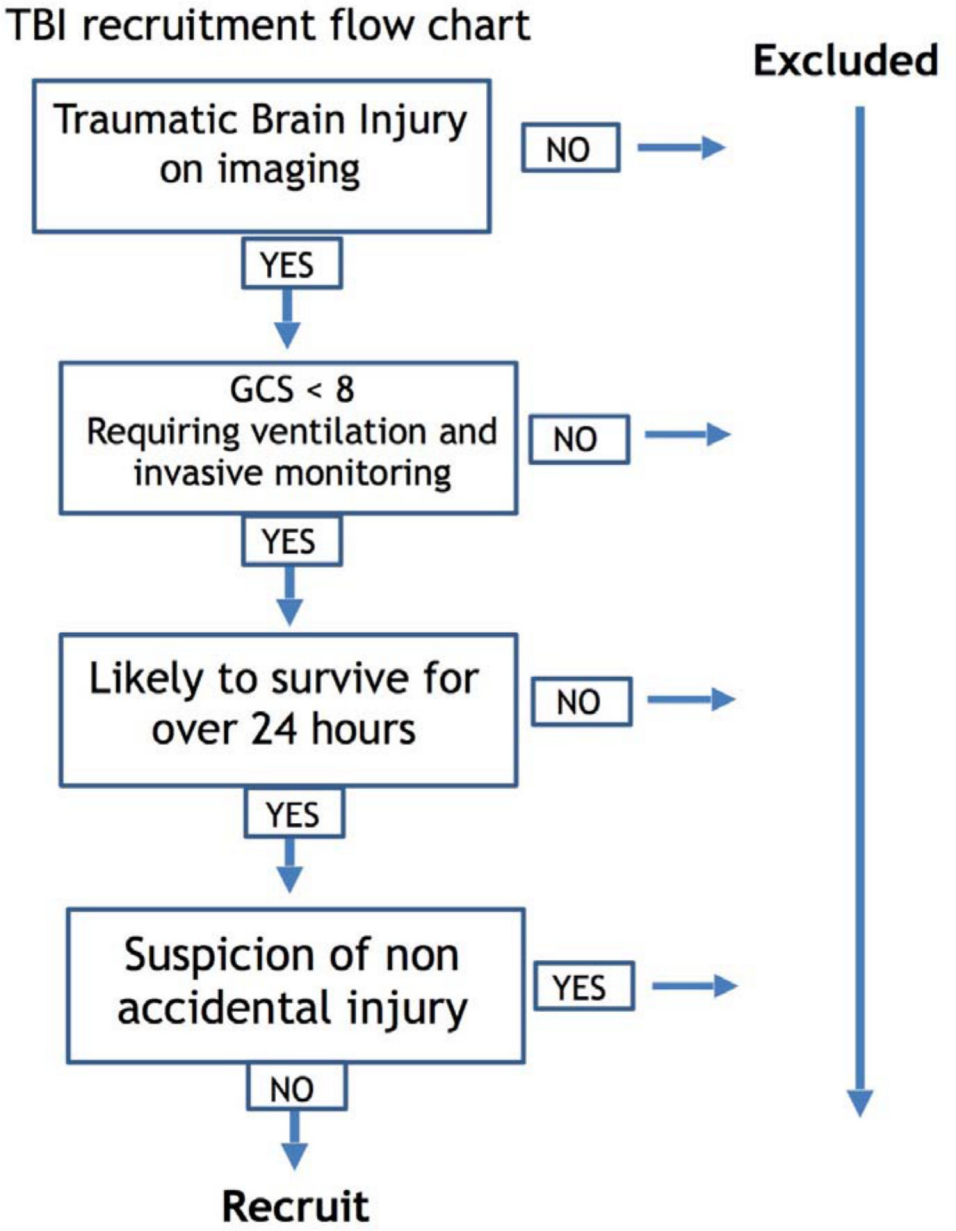
Flow chart demonstrating the recruitment of pediatric traumatic brain injury patients.

Inclusion criteria were as follows: 1) TBI -related pathology, confirmed on CT or MRI, 2) severe injury (GCS <8) failing to demonstrate significant early clinical improvement (i.e. poor neurology on sedation hold) and 3) requiring invasive monitoring of ICP and MAP. Patients were excluded if they were unlikely to survive for over 24 hours or there was any suspicion of non-accidental injury (NAI; Fig 1). Patients were managed according to current pediatric TBI guidelines [11] and followed the local protocol established in the pediatric intensive care unit (PICU; S1 Appendix). Patients were initially sedated, intubated, ventilated, and paralyzed. Interventions were aimed at keeping ICP < 20 mm Hg using a stepwise approach of head-up positioning, sedation/analgesia, muscle paralysis, moderate hyperventilation, ventriculostomy and osmotic agents. All patients were sedated and paralysed in the cohort however no patients received induced hypothermia or barbiturate coma. Only two patients received vasopressor support to maintain CPP (neither of which survived). No patients underwent decompressive hemicraniectomy.

### Data Acquisition and Analysis

**Fig 2:**
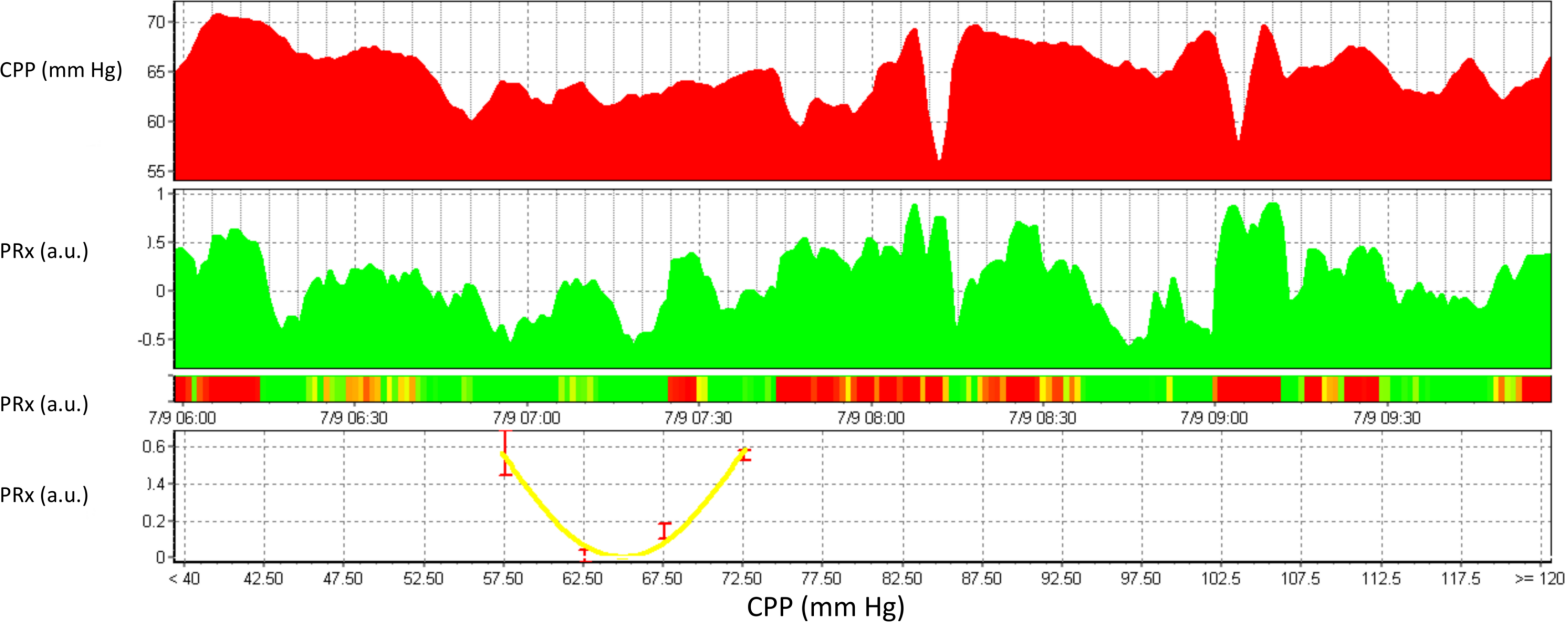
Example of a 4 hour epoch of multimodality monitoring signals in pediatric TBI. In this screenshot, CPP is shown in the top panel and the pressure reactivity index in the second panel over a 4 hour period from 06:00 to 10:00. In the third panel is a risk chart whereby a negative PRx (good autoregulation) is denoted by a grey colour, and a disturbed PRx (>0.3) is denoted in black. In two instances, CPP drops below 60 mm Hg. During these drops in CPP, PRx is deranged (black on the risk chart). On the bottom panel, CPP is plotted against PRx and a polynomial curve is fitted. The minimum of this curve is around 65 mm Hg, which would therefore indicate the optimal CPP at time point 10:00.

**Fig 3:**
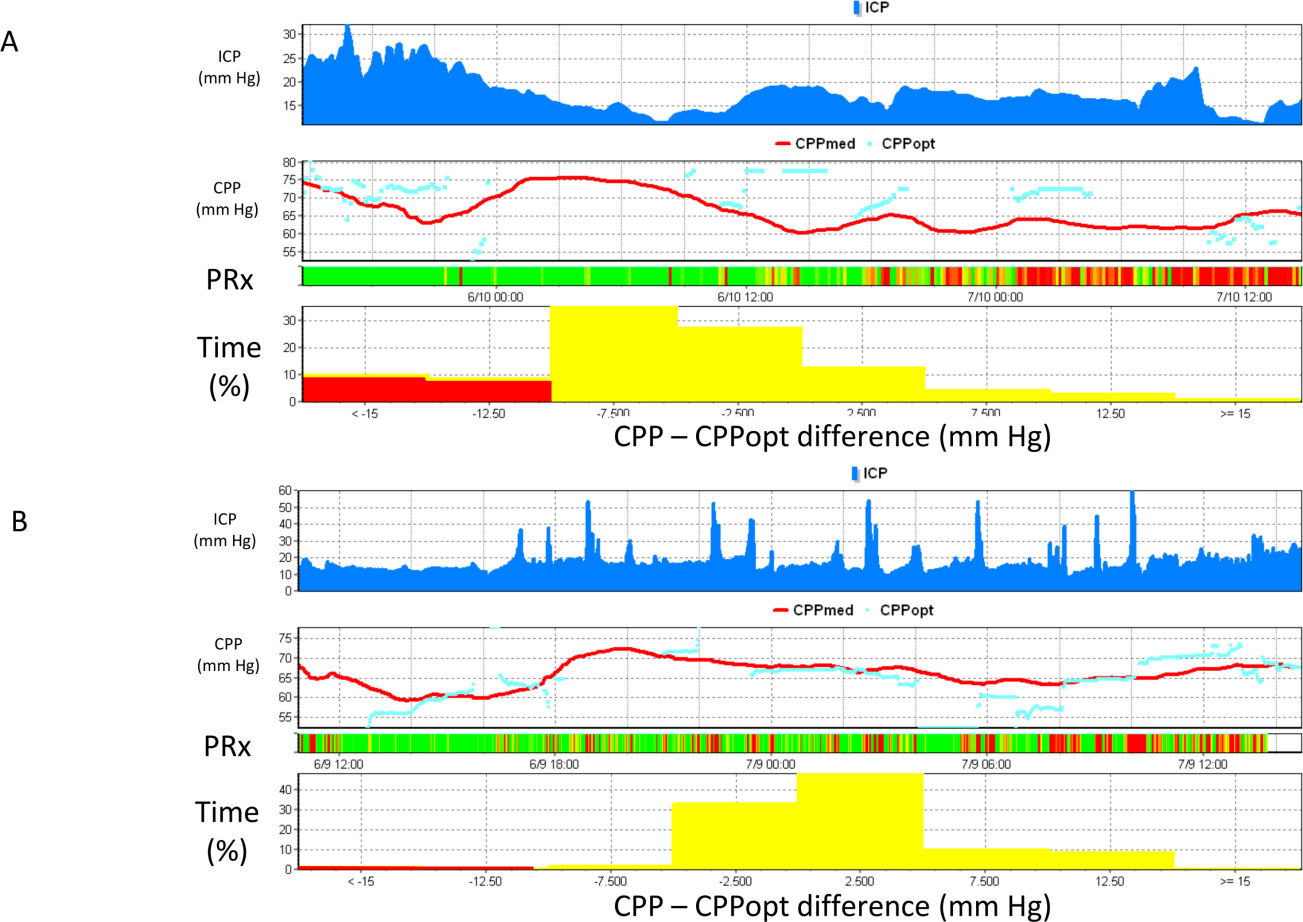
Real-time calculation of CPPoptimal in-vivo. Fig 3A is an example of a pediatric TBI patient. ICP is displayed in the top panel, followed by CPP (both the absolute CPP (line) and the calculated CPPopt (circles)), a risk chart of PRx and finally a histogram indicating the time spent at various distances from the calculated optimal CPP. Although this patients CPP was above 60 mm Hg for the whole of this recording, CPP was consistently below the calculated optimal CPP. This is depicted in the histogram which indicates that over this 2 day period, the patient spent almost 20% of time (expressed as a percentage of the total time CPPopt available) > 10 mm Hg below the instantaneous CPPopt. In the second day of this recording we see persistently disturbed PRx. This patient died three days after admission. Fig 3B shows an analogous example in another pediatric TBI patient. This patient demonstrated multiple plateau waves of ICP and a CPP between 60 and 70 mm Hg. Autoregulation as indicated by the PRx risk chart was mainly good. CPP was mainly close to the calculated optimal CPP as seen in both in the time series view (panel 2) and in the CPP-CPPopt time-histogram (bottom panel). This patient survived.

ICP was monitored with an intraparenchymal microsensor inserted into the right frontal cortex (Codman ICP Micro- Sensor, Codman & Shurtleff, Raynham, MA) and mean arterial pressure (MAP) was monitored in the radial or femoral artery with a zero calibration at the level of the right atrium (Baxter Healthcare CA, USA; Sidcup, UK). All signals were digitized using an A/D converter (DT9801, Data Translation, Marlboro, MA), sampled at a frequency of 100 Hz, and recorded using a laptop computer with ICM+ software (University of Cambridge, Cambridge Enterprise, Cambridge, UK, http://www.neurosurg.cam.ac.uk/icmplus). The same software was later used for the retrospective analysis of all stored signals (Figs 2 and 3). Time-averaged values of ICP, MAP, and CPP (CPP = MAP-ICP) were calculated using waveform time integration over 60-sec intervals. Cerebrovascular PRx was calculated as a moving Pearson correlation coefficient between 30 consecutive, 10-second averaged values of MAP and corresponding ICP signals (with 80% overlap of data) [7]. Averages over 10 seconds were used to suppress the influence of the pulse- and respiratory frequency wave components. A positive correlation between MAP and ICP in this frequency range is indicative of a passive cerebral vasculature and impaired autoregulation. Negative correlation between MAP and ICP at the same frequency is indicative of reactive vasculature and intact autoregulation [12,13,14]. PRx values greater than 0.2 indicate severely disturbed autoregulation and are associated with a poorer outcome in adults [7]. Artefacts were manually identified after data collection and excluded from further analysis.

Multi-modality monitoring was commenced at the nearest possible opportunity after the patient had been transferred to the tertiary setting and was terminated when sedation was lifted and the child either began to waken or died. The average time of data collection for the cohort was 3.5 days.

CPP optimal was continuously calculated as described previously [10]. Using the preceding 4 hours of data, PRx values were averaged into different observed CPP ranges spanning 5 mm Hg. An automatic curve fitting method (see Aries et al., [10] for details) was then applied to the CPP-PRx data to determine the CPP value with the lowest associated PRx value- CPPopt. This CPPopt value was updated every minute using a moving 4-hour time window. The algorithm was set so a CPPopt could be generated if at least 50% of the data points in the 4-hour window were available (i.e., with a minimum of 2 hrs of monitoring). An illustrative example from an individual patient is shown in Fig 3.

Values of measured variables from each patient were averaged (mean) over the whole monitoring period, so every patient was represented by one set of data containing MAP, ICP, CPP, and PRx. In individual patients, the differences between CPPopt and median CPP for the moving-window periods were calculated continuously (ΔCPP = median CPP–CPPopt). The percentage time each patient spent within different 5 mm Hg wide ranges of ΔCPP was calculated. The percentage time spent with a CPP more than 10 mm Hg below (ΔCPP< -10 mm Hg), more than 10 mm Hg above (ΔCPP> 10 mm Hg), or within 10 mm Hg (ΔCPP = -10 to 10 mm Hg) of CPPopt was calculated for each patient.

Clinical outcome was assessed by a clinician at discharge from ICU and at an outpatient clinic 6 months after the time of injury. The primary outcome measure was survival.

### Statistical Analysis

All physiological data are summarized as the medians with their associated interquartile range (IQR). Differences in physiological values between survivors and non-survivors were interrogated with the Mann-Whitney U-test. The significance level was set to 0.05, and all tests were two-tailed and unadjusted for multiple comparisons. All data analyses were performed on SPSS version 21.0 software (SPSS Inc., Chicago, IL).

## Results

The mean age at presentation was 6.25 years (range 3 months-13 years); the cohort was split equally between sexes (Table 1). 92% of patients were involved in a road traffic collision; 75% of which were car vs patient and 25% car vs car. One patient sustained a fall from a significant height. The Modified Marshall scores ranged from 1–5 with a mode of 2. Survival was identical at ICU discharge and at 6 months 8/12 (66%) of the cohort surviving the acute brain injury. Six survivors had a GOS of 5 with the remaining two children having a score of 4 and 3 respectively.

**Table 1:** Table of demographics of patients with traumatic brain injury included in the study. The statistical tests are all univariate and uncorrected for multiple comparisons.

	Survived (n = 7)	Non-surviors (n = 5)	p value
**Age, mean ± SD**	5.9 ± 3.9	8.2 ± 6.5	0.45
**Male (%)**	3 (43)	3 (60)	0.62
**Admission GCS, median (range)**	9 (3–13)	3 (3–9)	0.26
**Motor Score**	4 (1–6)	1 (1–4)	0.21
**Mechanism of injury**			
*Car vs Patient*	3 (43)	5 (100)	0.07
*Car vs Car*	3 (43)	0 (0)	0.03
*Fall from height*	1 (14)	0 (0)	0.24
**Pupils**			
*Reactive (%)*	5 (72)	2 (40)	0.1
*Fixed Unilaterally (%)*	1 (14)	2 (40)	0.45
*Fixed Bilaterally (%)*	1 (14)	1 (20)	1
**Hypoxia**	1 (14)	2 (40)	0.45
**Hypotension**	2 (28)	0 (0)	0.15
**Modified Marshall Score, (median)**	2	3	NA
**SAH on CT (%)**	5 (72)	4 (80)	0.8
**Epidural Mass (%)**	4 (58)	1 (20)	0.09
**Petechial Haemorrhages (%)**	3 (42)	1 (20)	0.6
**Obilteration of Basal Cisterns (%)**	2 (28)	3 (60)	0.3
**Mid-line shift (%)**	4 (58)	1 (20)	0.09
**Surgical Intervention**			
*External Ventricular Drain*	2 (28)	1 (20)	0.3
*Haematoma Evacuation*	2 (28)	0 (0)	0.15
*Decompression*	1 (14)	1 (20)	1
**Haemoglobin, mean ± SD**	7.9 ± 1.9	11.6 ± 3.1	0.01
**Glucose, mean ± SD**	11.4 ± 1.6	11.1 ± 3.9	0.9
**Glasgow Outcome Score, median (range)**	5 (3–5)	1 (1)	0.001

**Fig 4:**
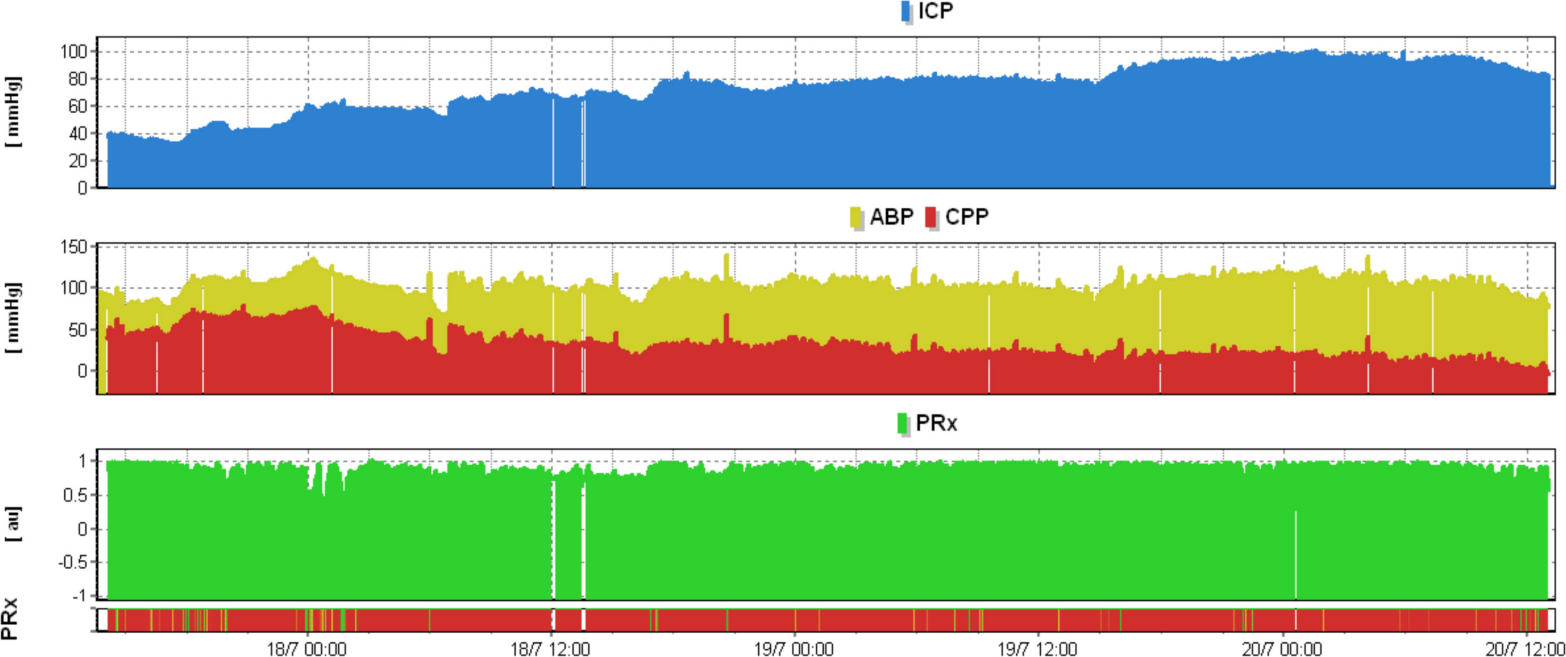
Severely impaired pressure reactivity index during refractory intracranial hypertension. High intracranial pressure was associated with persistently impaired pressure reactivity index as indicated by the solid red line (coded as black in this figure) on the PRx risk chart. In this situation, fluctuations in MAP are transmitted to ICP.

The median ICP was significantly lower in those who survived 13.1±3.2 mm Hg (median ± IQR) compared to those who did not 21.6±42.9 mm Hg (p = 0.003; Table 2). Additionally, in the survivor group patients had a raised ICP (> 20 mmHg) for only 9.7+9.8% of the time compared to 60.5±67.4% in non survivors (p = 0.003). There was a trend towards a lower arterial blood pressure (MAP) in those who survived 77.1±17.7 mm Hg compared to those who did not 93.0±24.2 mm Hg (p = 0.11). The pressure reactivity index (PRx) also provided significant outcome separation, with survivors having a median of 0.02±0.19 as compared to 0.39±0.62 in deceased patients (p = 0.02; Fig 4).

**Table 2:** Multimodality monitoring parameters in brain injured patients stratified by outcome at completion of brain monitoring. ICP–intracranial pressure, MAP–mean arterial pressure, CPP–cerebral perfusion pressure, CPPopt–optimal cerebral perfusion pressure, PRx–pressure reactivity index, RAP–cerebrospinal compensatory reserve.

	Survivors (n = 7)		Non-survivors (n = 5)		P-value
	Median	IQR	Median	IQR	Mann-Whitney
**ICP (mm Hg)**	13.07	3.23	21.64	42.90	0.003
**%time ICP > 20 mm Hg**	9.73	9.84	60.47	67.40	0.003
**MAP (mm Hg)**	77.07	17.69	93.00	24.23	0.11
**CPP (mm Hg)**	63.42	8.10	61.11	38.62	0.91
**CPPopt (mm Hg)**	63.68	8.94	66.45	18.70	0.48
**PRx (a.u.)**	0.02	0.19	0.39	0.62	0.02
**RAP (a.u.)**	0.64	0.27	0.46	0.43	0.11
**%time CPPopt available**	55.96	13.84	43.48	28.32	0.02
**Duration (hours)**	75.3	22.3	82.3	61.8	0.87
**%time CPPopt available**	55.96	13.84	43.48	28.32	0.02
**%time CPP-CPPopt < -10 mm Hg**	4.70	5.68	15.17	30.74	0.04
**%time CPP-CPPopt -10 to 10 mm Hg**	90.68	12.64	70.61	21.78	0.02
**%time CPP-CPPopt > 10 mm Hg**	5.09	10.03	11.62	16.9	0.76

The statistical tests are all univariate and uncorrected for multiple comparisons.

**Fig 5:**
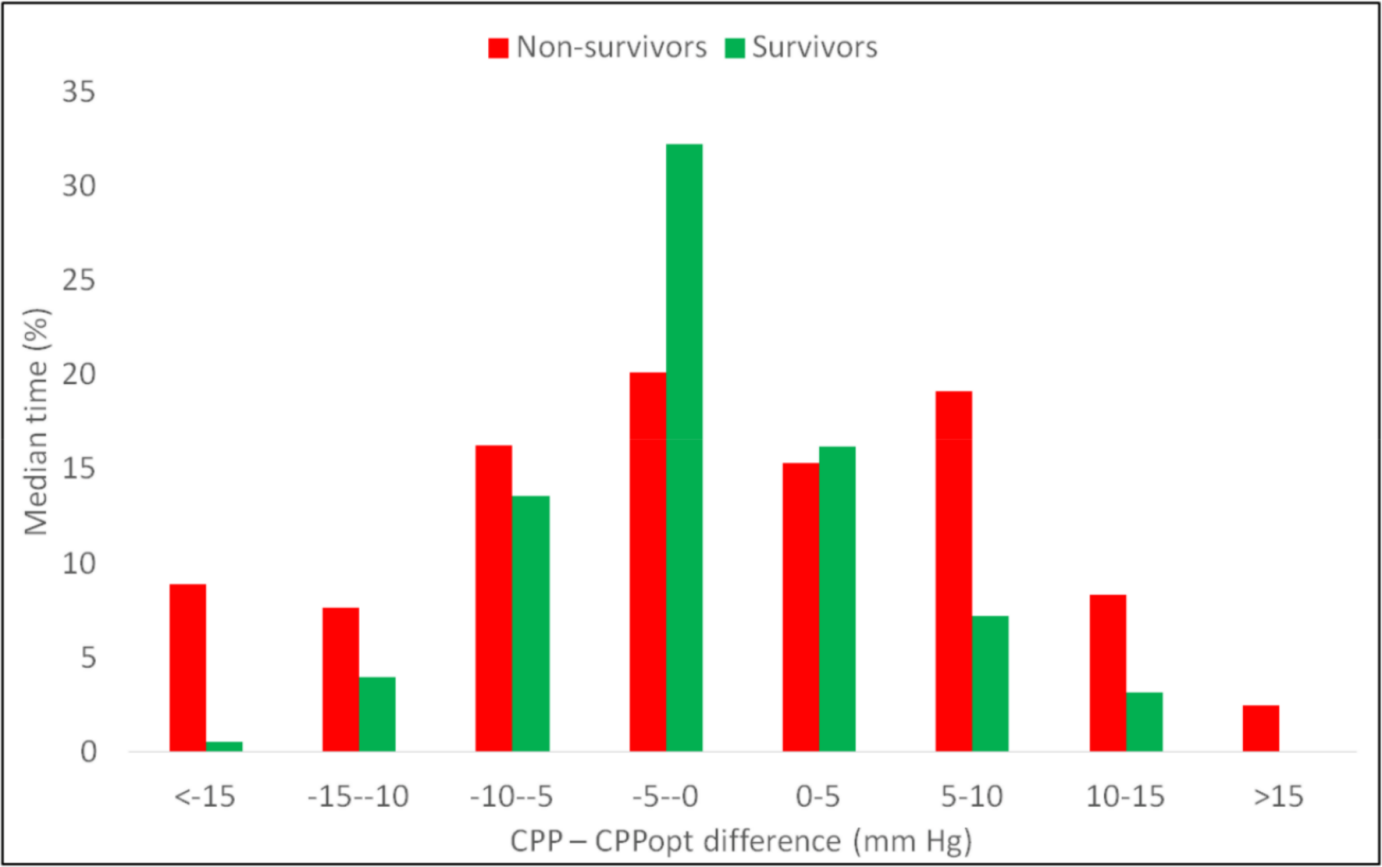
Time spent at different distances from CPPopt in non survivors (light grey) and survivors (dark grey). In those that survived the distribution of time spent across the various CPP-CPPopt (ΔCPP) ranges approximated a normal distribution. Most time was spent in the range between -10 and positive 10 mm Hg, while only a small proportion of time was spent less than -10 mm Hg or greater than 10 mm Hg from CPPopt. In non-survivors (light grey), more time is spent in the extreme CPP–CPPopt (ΔCPP) ranges. This is reflected in Table 2, those who did not survive spent significantly more time with their with their CPP more than 10 mm Hg below CPPopt (Mann-Whitney p = 0.04) and less time with their CPP within 10 mm Hg of the CPPopt (Mann-Whitney U test p = 0.02).

Although there was no evidence that mean cerebral perfusion pressure (CPP) over the whole monitoring period was different between those patients who survived versus those who died (p = 0.91) there were significant differences with regards to deviation from CPPopt (ΔCPP). Specifically, the duration for which the CPP deviated from CPPopt was significantly different in survivors (Example data Fig 3 and Results Fig 5). Also, in survivors, time spent with CPP lower than CPPopt by more than 10 mm Hg was as low as 4.7 ± 5.7% of the total time compared to 15.12 ± 30.74% in non-survivors (p = 0.04). Notably, the time spent with CPP greater than CPPopt by more than 10 mm Hg was also lower for survivors, 5.1 ± 10.0%, than in non-survivors, 11.6 ± 16.9% though the difference was not significant (p = 0.76). On the whole, the total time spent in the ±10mmHg zone around CPPopt was 90.7 ± 12.6% in survivors compared to 70.6 ± 21.8% in non-survivors (p = 0.02).

## Discussion

Our results support the argument proposed by Brady et al.,[5] that PRx can be used to delineate optimal ranges in CPP on an individual basis. Importantly, we have shown that CPPopt can be calculated in the pediatric population and may have a prognostic value. Moreover, we have identified that high ICP, high PRx and longer time spent with a sub-optimal CPP are all associated with higher mortality when analysed with a univariate model mirroring similar findings in adults [10].

Multi-modal monitoring has been used extensively in adult TBI to observe haemodynamic stability, limit secondary insults, obtain accurate neurological assessment and appropriately select patients for further investigation after acute brain injury [3,15]. But even though patient population studies have demonstrated that the incidence of hospitalisation and fatal brain injury is disproportionately high in children, little has been published on application of multi-modal monitoring in children [4,5].

The data presented here provides an important insight into the neurophysiology following acute brain injury in the pre-teenage years. The cohort has similar demographics to that reported by Brady et al., [5] and there are a number of important similarities to be observed in the two studies. The modified Marshall score was included in the demographics to demonstrate both the severity of injury and how likely the patients were to survive based on predictive scores in adults [16,17].

The assertion that ICP and CPP affect outcome in adults has been well established for over 60 years [18]. The rigidity of the cranium determines a limited ability to perfuse neural tissue when ICP is raised [19]. In adults, common practice is to augment arterial blood pressure in instances of raised ICP. However, not only marked elevations of CPP accelerate oedema leading to secondary intracranial hypertension [20, 21] but they can frequently contribute to systemic insult [22]. This is more frequently observed in patients who present with a reduced GCS as was the case in our cohort [10]. Although there is insufficient level I & II evidence to support the notion that uncontrolled ICP and CPP effects outcome in children after brain trauma, joint management of ICP and CPP is considered standard practice for managing pediatric patients with severe TBI [11].

Reassuringly, we confirmed in our cohort that high ICP is strongly associated with morbidity. But while Brady et al. [5] found a significant correlation between CPP and outcome this was not observed here. Instead, there was a significant relationship between outcome and the deviation from optimal CPP. This seems to be an important finding in this study. The concept of individualised CPP in adult population has been around for over a decade [8], and has recently been quoted as an optional strategy in the current TBI guidelines [3], however its applicability in children is still debated. While there have been no large studies to determine CPPopt in children Chambers et al., [23] proposed a critical CPP for age stratified populations in children. Specifically, in the age groups 2–6, 7–10 and 11–16 years of age good outcomes were associated with CPP values of 43, 54 and 58 mm Hg respectively [21]. Using ΔCPP values it was observed that when values of CPP deviate from optimum there is a relationship with regards to outcome and pressure difference. In particular, those who spent more time with CPP lower than CPPopt had a poorer outcome.

The findings here stress the importance of guiding TBI treatment using autoregulation indices in clinical practice. It may be interpreted that any difference between real and target CPP greater than 10 mmHg must be avoided. These preliminary observations support the need further investigation into the feasibility of CPPopt oriented therapy.

Additionally, it is of interest that the haemoglobin was significantly lower in patients who survived. Whilst the mechanism or interpretation of this is unclear, particularly with the low numbers involved this could potentially be linked to pre-hospital resuscitation with intravenous fluids, which if performed correctly can improve outcome [24].

In a similar fashion to previous reports, we have observed that cerebrovascular pressure reactivity provides a strong association with outcome. These findings are particularly interesting because unlike in adults, children are unlikely to have any pre-existing systemic vascular pathology that could result in impaired cerebrovascular pressure reactivity [16]. Therefore, it likely that in these children, that the impaired PRx was a consequence of the trauma.

### Limitations

An important limitation of this preliminary report is the small sample size. which precludes multivariate analysis, and in particular relationship between age, ICP and CPP optimal. Nevertheless, the principal finding of intracranial pressure, pressure reactivity status and deviation from CPPopt being important predictors of outcome is consistent with existing evidence in adults.

Further, CPPopt could only be calculated less than 60% of the monitoring period. This low yield is a consequence of the rigid criteria inherent in the curve fitting algorithm (See appendix of Aries et al., [10]), and may also be influenced by clinical or physiological events such as low ABP variability [24]. Techniques to improve the availability of CPPopt targets are essential if the CPPopt concept is to provide real-time clinical support and such techniques should be investigated in further studies. Finally, the study was a preliminary feasibility study and was not designed to measure the effects of critical care interventions such as surgical interventions, medication exposure (eg, barbiturates, hypertonic saline solution, or vasoactive agents).

## Conclusion

Our observations provide further evidence that multi-modality monitoring is useful in children with acute brain injury. ICP, PRx and ΔCPP appear to provide significant correlations with outcome. As such, a large multicenter, prospective study is required

## Supporting Information

S1 Appendix Local ICP protocol for Cambridge University Hospitals head injury management. (PDF)

## Abbreviations

MAP: mean arterial pressure
CPP: cerebral perfusion pressure
ICP: intracranial pressure
PICU: Pediatric Intensive Care Unit
PRx: Pressure reactivity index
CPPopt: Optimal CPP
TBI: Traumatic brain injury
