# Supplementary material for: Continuous Multimodality Monitoring in Children after Traumatic Brain Injury—Preliminary Experience
Source: PLoS One. 2016 Mar 15;11(3):e0148817. doi: 10.1371/journal.pone.0148817 (PMC4792535; doi:10.1371/journal.pone.0148817)
Supplement: S1 Appendix — (PDF) [file pone.0148817.s001.pdf]

Protocol for managing paediatric severe traumatic brain injury

TARGETS

- SpO_2 >97%, PaO_2 >9kPa, PCO_2 4.5-5 kPa
- Temp < 37°C, Glucose 4-7 mmol/l
- Serum Sodium > 140 mmol/l

ICP/CPP

Age (yr)	ICP (mm Hg)	CPP (mm Hg)
<3	5-15	40
4-7	15-20	40-50
>8	< 20	50-60

Stage A

- Nurse 30° head up
- Ensure no venous obstruction to neck
- Sedation:
    - Midazolam: 50-300 µg/kg/hr
    - Fentanyl: 1-3 µg/kg/hr *or*
    - Morphine: 20-80 µg/kg/hr
- Paralysis:
    - Atracurium: 300-600 µg/kg/hr *or*
    - Vecuronium: 50-100 µg/kg/hr
- Anticonvulsants: Phenytoin 15 mg/kg (depressed #, seizures)
- Antibiotics: none for CNS reasons unless discussed with neurosurgeons
- Ventilation: TV 6-8 ml/kg & rate to keep pCO_2 in target range, no hyperventilation

⇩

ICP/CPP targets not met?
Stage B

- 5% saline 2-4 ml/kg
  (can be repeated but plasma osm < 320) *or*
  Mannitol 20% 2 ml/kg
- Ventilation pCO2 ~ 4.5 kPa
- Hypothermia: temperature 35°C
- External ventricular drain if feasible
- Consider anticonvulsants if not already given

⇩

ICP/CPP targets not met?
Stage C

- Discuss with PICU consultant/ neurosurgery team and decide either
- Thiopentone 2 mg/kg/hr to achieve burst suppression (cfm/ continuous EEG) or
- Consider decompressive craniectomy

Patients Details

SPINE
Consider unstable until cleared by the neurosurgeons
Use sandbags/tape/collar to immobilise

ICP Target:

……………………………mm Hg

Signature: ……………………………

Date:…………………………………..

CPP Target:

……………………………mm Hg

Signature: ……………………………

Date:…………………………………..

If targets not met:

- Consider repeat CT scan
- Consider recalibrating the probe

Neurosurgical bleep:	156-0358
PICU on-call bleep:	152-001

Shruti Agrawal, Matthew Garnett March 2014
